# The higher the farther: distance-specific referential gestures in chimpanzees (*Pan troglodytes*)

**DOI:** 10.1098/rsbl.2017.0398

**Published:** 2017-11-15

**Authors:** Chloe Gonseth, Fumito Kawakami, Etsuko Ichino, Masaki Tomonaga

**Affiliations:** 1 Primate Research Institute, Kyoto University, Inuyama 484-8506, Japan; 2 Kyoto University Institute for Advanced Study, Kyoto 606-8501, Japan

**Keywords:** chimpanzees, referential communication, deixis, distance-specific oral and manual gestures

## Abstract

Referential signals, such as manual pointing or deictic words, allow individuals to efficiently locate a specific entity in the environment, using distance-specific linguistic and/or gestural units. To explore the evolutionary prerequisites of such deictic ability, the present study investigates the ability of chimpanzees to adjust their communicative signals to the distance of a referent. A food-request paradigm in which the chimpanzees had to request a close or distant piece of food on a table in the presence/absence of an experimenter was employed. Our main finding concerns the chimpanzees adjusting their requesting behaviours to the distance of the food such that higher manual gestures and larger mouth openings were used to request the distant piece of food. To the best of our knowledge, this is the first study to demonstrate that chimpanzees are able to use distance-specific gestures.

## 1. Background

Referential signals, such as manual pointing or deictic words, are a crucial component of human communication as they play a central role in social skills and language acquisition [1,2]. These signals are intended to direct the attention of others to specific external entities and to share attention, feelings, and thoughts about them [3]. Additionally, some of these deictic signals enable humans to efficiently and accurately locate a referent in the environment for one's own personal benefit or the benefit of another. Individuals can divide space into different areas, usually according to near versus far [4], resorting to appropriate distance-specific linguistic and gestural units. Thus, the distance of a referent is encoded at a lexical (high) level of language processing (using specific lexical units, i.e. deictic words) [5] as well as at a motor (low) level of language processing (using specific gestural units, such as articulatory components, or manual pointing, the hand being seen here as a linguistic tool in itself) [6]. Gonseth *et al*. [6,7] demonstrated that humans exhibit greater amplitudes during manual pointing towards distant than close objects and that articulatory properties of vocal pointing also vary with the referent's distance. Although participants in that study had to use a same manual gesture (an index finger pointing) and a same deictic word (‘there’) to designate a close or a distant luminous target, they showed larger manual pointing, in terms of index finger trajectory, and larger lip openings to designate the distant one. Thus, distance encoding is a robust feature of the human referential system, present in all aspects of multimodal pointing.

Identifying this encoding in the signalling behaviours of our closest evolutionary relatives could provide valuable information regarding the emergence and mechanisms of deixis. Although referential abilities have been well-studied in non-human primates, spatial deixis in these species remains understudied. For instance, it is well-known that both monkey and ape referential gestures show some language-like properties [8–12], but it is unclear whether these signals can be adjusted to the spatial properties, here the distance, of a referent (see [13], investigating other spatial features). To our knowledge, no studies have investigated this issue; however, Roberts *et al*. [14] mentioned a possible distance encoding mechanism in the gestures of a single language-trained chimpanzee. The present study employed a food-request paradigm in which chimpanzees had to request a close or distant piece of food on a table in the presence/absence of a human interlocutor to investigate the ability of chimpanzees to adjust their signals to the distance of a referent. Chimpanzees' gestures being intentional [9–11,15], they are expected to produce primarily visual signals when the interlocutor is present and primarily auditory signals when the interlocutor is away (here, out of sight). More crucially, if distance encoding is already present in chimpanzees, distance-specific oral and manual gestures should be observed.

## 2. Methods

A description of the methods is provided in the electronic supplementary material.

**Figure 1.**
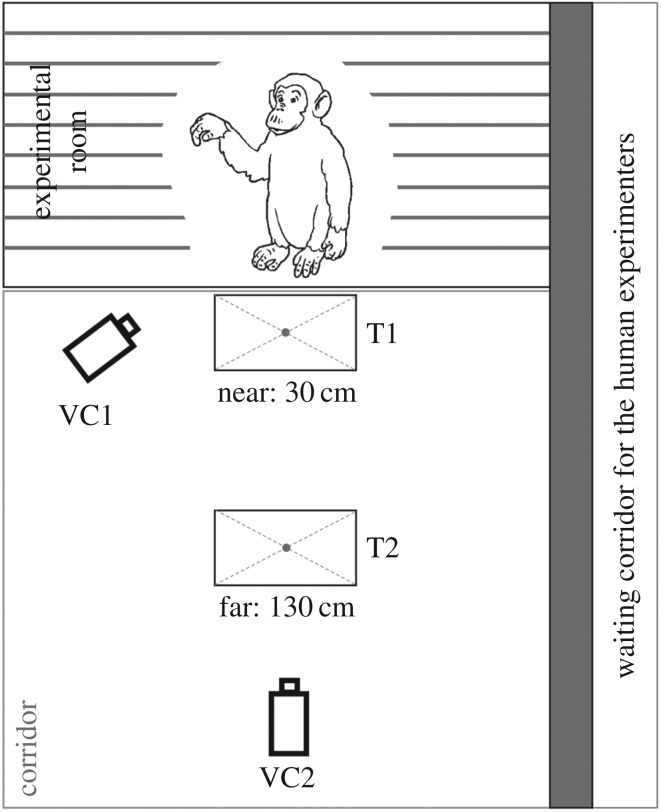
Experimental setting. Two tables (T1 and T2) were placed in the corridor in alignment but at different distances from the railings (‘near’ and ‘far’). Two video cameras (VC1 and VC2) recorded all experimental events.

Eight chimpanzees living at the Primate Research Institute of Kyoto University, Japan [16] were individually tested in an indoor room, separated from experimenters by railings made of metallic bars. Two identical tables (T1 and T2) were placed in the corridor on the experimenters' side of the railings in alignment but at different distances (‘near’ and ‘far’; figure 1). Two video cameras (VC1 and VC2) recorded all sessions.

The requesting behaviours of the chimpanzees were tested under two conditions. In the test (‘with-human’) condition, the first experimenter (E1) placed a piece of banana on one table and left the area. Then, the second experimenter (E2) approached the chimpanzee and engaged her/him as soon as she/he produced the first request behaviour or after 5 s. The chimpanzees were given the food by E2 within 15 s of their first request regardless of their behaviour during the trial. The control (‘alone’) condition was similar to the test condition except that E2 was absent, as a way to confirm that the gestures produced in the test condition were directed to the interlocutor rather than induced by the sole presence of the food. The chimpanzees were alone while waiting for the food and were given the food by E1 15 s from her departure. Each chimpanzee performed 10 test sessions and five control sessions, up to one per day. Each session comprised eight trials, including four trials with the food placed on the ‘near’ table.

All signals produced by the chimpanzees were coded from the video data and categorized into manual requesting gestures (begging and pointing gestures through the railings), attention-getting behaviours (clapping or banging), and others (e.g. silent mouth opening). The modalities of these signals were also categorized into visual or silent gestures (e.g. manual pointing), auditory signals (e.g. vocalizations), and audio-visual signals (e.g. attention-getting behaviours). Eventually, auditory and audio-visual signals were combined into a single ‘audio-visual’ category, since only 0.4% of the signals produced here were vocalizations. Manual pointing and begging and silent mouth openings were further coded in greater detail such that the height of the hand (in cm) and the aperture of the mouth (‘small’ or ‘large’) were noted (both measurements were based on the railings' thickness and gap). Systematic variations in the qualitative features of these gestures based on the distance of the referent were expected; more specifically, spatially extended gestures were expected when requesting distant pieces of food.

## 3. Results and discussion

Descriptions of the statistical analyses and results are provided in the electronic supplementary material.

**Figure 2.**
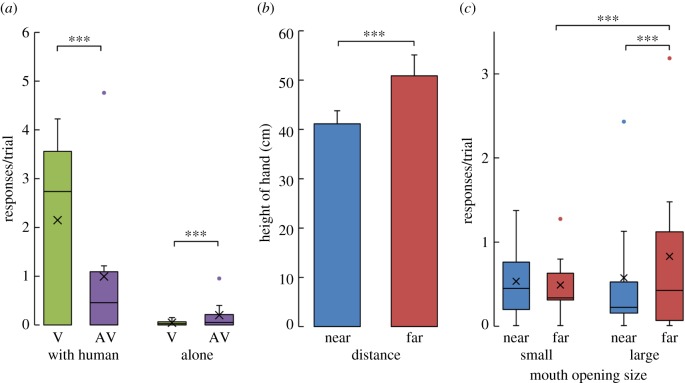
(*a*) Average number of visual (V) and audio-visual (AV) signals per trial depending on the condition (‘with human’ versus ‘alone’). (*b*) Average height of the hand (in cm) per trial depending on the distance of the food (‘near’ versus ‘far’). (*c*) Average number of small and large mouth openings per trial depending on the distance of the food (‘near’ versus ‘far’). The ‘X’ represents the mean, the horizontal lines the median, and the dots the outliers.

Figure 2*a* shows the mean number of visual (V) and audio-visual (AV) signals per trial depending on the condition. Chimpanzees produced more gestures under the test (‘with human’) than under the control condition (‘alone’; generalized linear mixed model (GLMM), *Z* = 8.620, *p* < 0.001). Furthermore, they used more visual than audio-visual signals in the presence of the experimenter (*Z* = 11.089, *p* < 0.001) but more audio-visual than visual signals in the absence of the experimenter (*Z* = 3.513, *p* < 0.001). Unsurprisingly, the chimpanzees produced intentional and communicative signals rather than merely food-associated signals.

More importantly, the chimpanzees adjusted their manual and non-vocal oral gestures according to the distance of the referent [7]. Figure 2*b* shows the mean height of the hand (in cm) per trial as a function of the distance of the food (‘near’ versus ‘far’). A significant effect of distance on the height of the hand (GLMM, *Z* = 9.346, *p* < 0.001) was observed, indicating that the chimpanzees used higher manual gestures to request a distant piece of food. Figure 2*c* shows the mean number of ‘small’ and ‘large’ mouth openings per trial depending on the distance of the food (‘near’ versus ‘far’). The chimpanzees produced more large mouth openings when the food was far compared with when it was near (GLMM, *Z* = 3.819, *p* < 0.001), but they also produced more large openings than small ones when the food was far (*Z* = 5.222, *p* < 0.001). Interestingly, this effect was not significant for either the small opening or when the food was close. This suggests that, for a close referent, manual distance encoding is sufficient, whereas chimpanzees might need to provide more information via the oral system for a farther referent.

In summary, the present study demonstrated that chimpanzees adjust their signals to both the presence/absence of an interlocutor and, more importantly, the distance of the referent. The chimpanzees were able to take the presence of another individual into account and tailor their signals in an appropriate manner. Furthermore, to the best of our knowledge, this is the first study to demonstrate that chimpanzees are able to use distance-specific gestures. This ability to gesturally distinguish a close from a far space is quite similar to the sophisticated use of deictic words and gestural pointing used by humans and suggests a close connection between the manual and oral systems. In the present study, chimpanzees used spatially extended oral and manual gestures to request a distant piece of food, a phenomenon observed in human language at a gestural level as well as a linguistic level [5–7]. More specifically, humans use spatially extended oral and manual gestures to designate a distant object by exhibiting a larger mouth opening and greater manual gesture amplitude for a farther object [6,7]. This pattern is consistent with the most common phonological pattern for the use of deictic words throughout the world (see [6] for a review): for example, open vowels, such as the /ɛ/ in ‘there’, are used for distant deictic targets, whereas close vowels, such as the /i/ in ‘here’, are used for close deictic targets. This example of phonosymbolism (a non-arbitrary relationship between phonetics and semantics), in conjunction with the gestural encoding of distance in both the oral and manual gestures, indicates a close relationship between linguistic structures and communicative gestures in human language. In other words, the universal tendency to use open/closed vowels for distant/close objects might be rooted in a general motor behaviour. The present results suggest that both deictic words and gestures may have emerged from highly symbolic oral and manual gestures (spatially extended gestures for a farther reference), progressively integrated into language phonology (open vowels). The presence of motivated combinations between phonetics and semantics would have then facilitated the emergence of vocabulary by constraining the manner in which words were first mapped onto referents. The distance encoding mechanism requires further investigation in chimpanzees and other primate species that are more and less phylogenetically distant from humans, both in captivity and the wild, to provide novel evidence for the multimodal and phonosymbolic emergence of language.

## Supplementary Material

Detailed descriptions of the methods, statistical analyses, and results

## Supplementary Material

Individual data (visual and audio-visual signals; height of the hand; mouth opening)

## Data accessibility

Coded data are available as electronic supplementary material.

## References

[1] Arbib MA 2005 From monkey-like action recognition to human language: an evolutionary framework for neurolinguistics. Behav. Brain Sci. 28, 105–167. (10.1017/S0140525X05000038) 16201457

[2] Tomasello M, Carpenter M, Call J, Behne T, Moll H 2005 Understanding and sharing intentions: the origins of cultural cognition. Behav. Brain Sci. 28, 675–691. (10.1017/S0140525X05000129) 16262930

[3] Liebal K, Waller BM, Burrows AM, Slocombe KE 2013 Primate communication: a multimodal approach. Cambridge, UK: Cambridge University Press. 10.1177/147470491301100305 PMC10480985 23864293

[4] Kemmerer D 1999 ‘Near’ and ‘far’ in language and perception. Cognition 73, 35–63. (10.1016/S0010-0277(99)00040-2) 10536223

[5] Diessel H 2005 Distance contrasts in demonstratives. In World atlas of language structures (eds Haspelmath M, Dryer M, Gil D, Comrie B), pp. 170–173. Oxford, UK: Oxford University Press.

[6] Gonseth C, Vilain A, Vilain C 2013 An experimental study of speech/gesture interactions and distance encoding. Speech Commun. 55, 553–571. (10.1016/j.specom.2012.11.003)

[7] Gonseth C 2013 Multimodalité de la communication langagière humaine: interaction geste/parole et encodage de la distance dans le pointage [Multimodality of linguistic communication: gesture/speech interaction and distance encoding in pointing tasks]. Doctoral dissertation Retrieved from theses.fr database (accession number 2013GRENS011). (In French with English abstract.)

[8] Call J, Tomasello M 2007 The gestural communication of apes and monkeys. New York, NY: Taylor & Francis Group, Lawrence Erlbaum Associates.

[9] Hobaiter C, Byrne RW 2014 The meanings of chimpanzee gestures. Curr. Biol. 24, 1596–1600. (10.1016/j.cub.2014.05.066) 24998524

[10] Leavens DA, Hopkins WD 1998 Intentional communication by chimpanzees: a cross-sectional study of the use of referential gestures. Dev. Psychol. 34, 813–822. (10.1037/0012-1649.34.5.813) 9779730 PMC2080769

[11] Hostetter A, Cantero M, Hopkins W 2001 Differential use of vocal and gestural communication by chimpanzees (*Pan troglodytes*) in response to the attentional status of a human (*Homo sapiens*). J. Comp. Psychol. 115, 337–343. (10.1037//0735-7036.115.4.337) 11824896 PMC2080764

[12] Bourjade M, Meguerditchian A, Maille A, Gaunet F, Vauclair J 2014 Olive baboons, *Papio anubis*, adjust their visual and auditory intentional gestures to the visual attention of others. Anim. Behav. 87, 121–128. (10.1016/j.anbehav.2013.10.019)

[13] Hopkins W, Wesley M 2002 Gestural communication on chimpanzees (*Pan troglodytes*): the influence of experimenter's position on gesture type and hand preference. Laterality 7, 19–30. (10.1080/13576500143000113) 15513185 PMC2175393

[14] Roberts AI, Vick SJ, Roberts SG, Menzel CR 2014 Chimpanzees modify intentional gestures to coordinate a search for hidden food. Nat. Commun. 5, 3088 (10.1038/ncomms4088) 24430433 PMC4350813

[15] Leavens DA, Russell JL, Hopkins WD 2010 Multimodal communication by captive chimpanzees (*Pan troglodytes*). Anim. Cogn. 13, 33–40. (10.1007/s10071-009-0242-z) 19504272 PMC2797826

[16] Matsuzawa T, Tomonaga M, Tanaka M 2006 Cognitive development in chimpanzees. Tokyo, Japan: Springer.

