# Supplementary material for: The higher the farther: distance-specific referential gestures in chimpanzees (Pan troglodytes)
Source: Biol Lett. 2017 Nov 15;13(11):20170398. doi: 10.1098/rsbl.2017.0398 (PMC5719372; doi:10.1098/rsbl.2017.0398)
Supplement: Detailed descriptions of the methods, statistical analyses, and results [file rsbl20170398supp1.docx]

**The higher the farther: Distance-specific referential gestures in chimpanzees (*Pan troglodytes*)**

Chloe Gonseth^1*^, Fumito Kawakami^2^, Etsuko Ichino^1^, Masaki Tomonaga^1^

^1^Primate Research Institute, Kyoto University, Inuyama, 484-8506, Japan

^2^ Kyoto University Institute for Advanced Study, Kyoto, 606-8501, Japan

[^*^chloe.gonseth@gmail.com](mailto:*chloe.gonseth@gmail.com)

Appeared in *Biology Letters*

**Supplementary Materials**

Detailed descriptions of the methods, statistical analyses, and results.

1. **Methods**
2. *Participants*

Eight chimpanzees, living in a group of 12 individuals (three males and nine females, age range: 14–48 years), participated in the present study between November, 2014 and April, 2015: Ai (female, born in 1976, ID Number for the Great Ape Information Network [GAIN-ID], https://shigen.nig.ac.jp/gain/, [1]: C-0434), Akira (male, born in 1976, GAIN-ID: C-0435), Chloe (female, born in 1980, GAIN-ID: C-0441), Mari (female, born in 1976, GAIN-ID: C-0274), Pal (female, born in 2000, GAIN-ID: C-0611), Pendesa (female, born in 1977, GAIN-ID: C-0095), Popo (female, born in 1983, GAIN-ID: C-0438), and Puchi (female, born in 1966, GAIN-ID: C-0436). The other four chimpanzees, two males (Gon, born in 1966, GAIN-ID: C-0437, and Ayumu, born in 2000, GAIN-ID: C-0608) and two females (Pan, born in 1983, GAIN-ID: C-0440, and Cleo, born in 2000, GAIN-ID: C-0609), could not participate in this study, being easily anxious when separated from their social group. Since our study required individual testing, we decided not to include them in our sample. At the time of testing, all individuals had access to environmentally enriched outdoor (770 m^2^) and attached indoor compounds [2], notably equipped with climbing frames, ropes, hammocks, and/or streams and various species of trees. Chimpanzees had unlimited access to water and were fed, throughout the day (including three main meals), with a wide variety of fresh fruits and vegetables, as well as nutritionally balanced biscuits. These chimpanzees had participated in various previous studies, including those that employed computer-controlled tasks [3, 4] and tasks with a face-to-face design [5].

1. *Setting*

Each chimpanzee was temporarily separated from his/her group and tested individually in an indoor room (approximately 3.5 m wide × 5.7 m deep × 5 m high) that was separated from the experimenter by metallic railings (2 cm in diameter, 5 cm apart from each other). Prior to each test session, two tables (37 × 57 × 72 cm) were placed in front of the experimental room (see Figure 1 in the main text), and the chimpanzees were instructed to sit on a platform (2.5 m wide × 65 cm deep × 70 cm high) in front of the tables; they were free to move around the room during the experiment. The centre of the first table was placed 30 cm from the railings (“near” space; see [6] for a definition), which was theoretically within arm reach of the chimpanzees, whereas the centre of the second table was placed 130 cm from the railings (“far” space; see [6] for a definition), which was out of arms reach and as far as allowed by the dimensions of the corridor. Two video cameras (Sony Handycam HDR-CX720V and HDR-PJ800) were placed in the corridor in front of the experimental room to record all experimental events.

1. *Procedure*

The requesting behaviours of the chimpanzees were assessed under two conditions. Under the test condition (“with human”), the first experimenter (E1) placed a piece of banana (approximately 3 cm long) on one of the two tables and then left the area after having made sure that the chimpanzee was paying attention to the food. The second experimenter (E2) then stood 65 cm away from the chimpanzee between the two tables while ignoring the food and engaged the chimpanzee as soon as he/she produced a first request behaviour or after 5 s. The chimpanzees were given the food by E2 within 15 s of their first request regardless of their behaviour during the trial. In order to not reinforce any particular behaviours, but also to create a proper interaction while maintaining the chimpanzees’ interest in the task, E2 behaved as naturally as possible while showing similar behaviours across sessions and individuals. For that purpose, E2 used only a few simple Japanese phrases (e.g. “*Nani ga hoshii desu ka*“/“*What do you want?*”), tried to follow the eye gaze of the chimpanzees, and carefully gave the food at the same position through the railings, independently from the food position during the trials. The control condition (“alone”) was similar to the test condition except that E2 was absent, as a way to confirm that the gestures produced in the test condition were directed to the interlocutor rather than induced by the sole presence of the food (and that would thus translate excitement rather than actual request). Thus, the chimpanzees were alone while waiting for the food that was given to them by E1 15 s after her departure. When not with the chimpanzees, E1 and/or E2 were waiting in a corridor next to the experimental room (see Figure 1 in the main text) and were not visible to the chimpanzees. The assignment of the researchers as E1 or E2 was determined by their relative familiarity with the individuals; however, both researchers were familiar with the chimpanzees as they were closely involved in their daily care and feeding. Each chimpanzee performed 10 test sessions and five control sessions, up to one per day, during weekday afternoons at 3 P.M. Each session comprised eight 15-s trials in which four trials had the food placed on the “near” table. The inter-trial interval was set at 30 s, and the order of exposure to the two conditions was randomised across sessions and chimpanzees. The position of the food (“near” vs. “far”) was also randomised across trials.

1. *Data scoring*

One of the authors (CG, main coder) semi-blindly coded the 960 trials (15 sessions × 8 trials × 8 chimpanzees) performed in the study using ELAN software (<https://tla.mpi.nl/tools/tla-tools/elan/>). In other words, even though this experimenter was aware of the condition under which the behaviours were produced (with or without a human interlocutor), this experimenter was not aware of the distance of the referent, tables, or food.

All communicative signals produced by the chimpanzees were coded as follows: manual requesting gestures (i.e. begging and pointing gestures), attention-getting behaviours (i.e. audible signals such as clapping or banging that could be produced either with the hands or feet), or others (i.e. silent mouth openings, presentation of the genitalia, and head movements). Additionally, the modalities of the behavioural responses were coded either as visual or silent gestures (i.e. manual pointing), auditory signals (i.e. vocalisations that were considered to be purely auditory signals even though they provided some visual information), or audio-visual signals (i.e. any audible gesture conveying both visual and auditory information, such as attention-getting behaviours or repeated manual pointing produced between two metallic bars). Note that for repeated gestures, which usually included banging or pointing produced with a moving hand or foot, a new occurrence was scored each time the individual changed the position of the effector. Note that the key criteria for intentionality (see [7], among others) were used to distinguish between actual “communicative gestures” and “non-communicative actions” (i.e. meaningless actions). Thus, to be considered truly communicative, the gestures had to be produced with the chimpanzees looking at the interlocutor and/or alternating their eye gaze between the food and the interlocutor (on the contrary, the meaningless “actions” were usually produced while looking away, at the railings, or at their own body parts). Chimpanzees should also show persistence and/or elaboration in case of communicative failure.

Requesting manual and non-vocal oral gestures (i.e. silent mouth openings produced without any vocalisations) were coded in greater detail. To be considered as manual pointing or begging, a gesture must have started with an extension of the arm (either complete or partial because the gestures were constrained by the railings) towards the food with the fingers and palm in line with the arm and facing either up (begging) or down (pointing) [7]. Both indexical and whole-hand pointing gestures were considered to be manual pointing (see [7] for a detailed explanation). For both manual pointing and begging, the height of the hand was coded. To achieve this, each bar of the railings was numbered from 1 to 24, and the height of the hand corresponding to the number of the bar on which the hand was placed during the gesture hold was recorded (see [8] for a definition of the different gesture phrases); the bar numbers were later converted into cm.

To be considered as requesting non-vocal oral gestures, mouth openings had to be maintained and directed towards the experimenter and/or the food without any vocalisations. Note that we use the term “oral” rather than “oro-facial” gestures here because we focussed on the lips only (other facial components, such as eyebrows, or facial expressions, were not taken into account). For each of the mouth openings, the aperture of the mouth was coded as “small” or “large”. Note that the chimpanzees produced most of their mouth openings against the railing bars; thus, these bars were used to code the openings: a small opening corresponded to one or less than one bar, whereas a large opening corresponded to more than one bar and up to three bars (i.e. the maximum aperture).

1. *Statistical analysis*

The total number of signals per session and the total number of signals in each response category (i.e. visual, auditory, and audio–visual) were calculated for each condition to test the intentional nature of the communicative signals. The chimpanzees produced a total of 2841 signals that consisted of 1844 visual signals (64.9%), 985 audio–visual signals (34.7%), and 12 auditory signals (0.4%); the auditory and audio–visual signals were combined into a single “audio-visual” category. We applied a general linear mixed model (GLMM) using the “glmer” function in the library “lmer” [9] and “lmer4Test” [10] in R 3.2.2 with condition (“with human” vs. “alone”), distance (“near” vs. “far”), and response modality (“visual” vs. “audio-visual”) included as fixed effects and participant and session included as random effects. A two-way interaction term was included in the model, a Poisson distribution was set as the error distribution, and the obtained parameters were compared using post hoc Z tests. The chimpanzees were expected to produce more signals, primarily visual ones, when the interlocutor was present; they were expected to produce primarily auditory signals when the interlocutor was absent.

The mean height of the hand per session under the “with-human” condition was calculated for each distance to test for a possible distance that would encode the manual requesting gestures; data from one female chimpanzee (Mari) were excluded due to the absence of manual gestures. A GLMM (lmer function with Gaussian error distributions) was conducted with distance (“near” vs. “far”) as a fixed effect and participant and session as random effects. The obtained parameters were compared with post hoc Z tests using “lmer4Test”, and manual pointing and begging were combined into a single category (i.e. requesting gestures). Systematic variations in the qualitative features of the requesting gestures were expected depending on the distance of the referent; more specifically, it was expected that spatially extended gestures would be performed when requesting distant pieces of food.

The total numbers of ”small” and ”large” mouth openings per session under the “with-human” condition were calculated for each distance to test for a possible distance that would encode oral gestures. A GLMM was conducted using the “glmer” function with distance (“near” vs. “far”) and opening (“small” vs. “large”) as fixed effects and participant and session as random effects. A two-way interaction term was included in the model, a Poisson distribution was set as the error distribution, and the obtained parameters were compared with post hoc Z tests. Systematic variations in the qualitative features of oral gestures were expected depending on the distance of the referent; more specifically, it was expected that spatially extended gestures would be performed when requesting distant pieces of food.

1. *Reliability*

For reliability purposes, 15% of the video data were assigned to a novel observer, which resulted in a total of 18 videos (144 experimental trials) that were randomly chosen across participants and sessions. The novel observer coded all communicative signals produced by the chimpanzees, the response category of the signals (visual, auditory, or audio–visual), the height of the hand for manual pointing and begging (from 1 to 24), and the mouth aperture for each silent mouth opening (“small” vs. “large”). Cohen’s kappa was used to assess reliability; the coefficients exceeded chance (p < .001) for each response category, height of the hand, and mouth aperture (κ = .82, .95, .83, and .86 for visual signals, auditory signals, audio–visual signals, height of the hand, and mouth aperture, respectively).

1. **Results**
2. *Intentional nature of chimpanzee communicative signals*

Figure 2*a* (in the main text) shows the mean number of visual (V) and audio-visual (AV) signals per trial depending on the condition. There was a main effect of the condition (GLMM, Z =8.62, P < 0.001), which indicates that the chimpanzees produced more signals in the presence of the interlocutor than in the absence of the interlocutor. Furthermore, they used more visual than audio–visual signals in the presence of the experimenter (Z = 11.089, P < 0.001) but more audio–visual than visual signals in the absence of the experimenter (Z = 3.513, P < 0.001). These results unsurprisingly demonstrate that the chimpanzees produced intentional and communicative signals rather than merely food-associated signals. Note that a main effect of distance was observed (Z = 2.349, P = 0.019) under the test condition (“with human”) such that chimpanzees produced more visual signals when the food was far rather than near. It would be interesting to analyse these signals more precisely to determine whether they were produced in combination or independently from one another. Multimodal signalling could indeed be another way to encode distance information.

1. *Distance-specific signals in chimpanzees*

The present study produced evidence that chimpanzees, much like human adults and children, adjust their manual and non-vocal oral gestures according to the distance of the referent [11]. Figure 2*b* (in the main text) shows the mean height of the hand (in cm) per trial as a function of the distance of the food (“near” vs. “far”). There was a significant effect of distance on the height of the hand (GLMM, Z = 9.346, P < 0.001) but not on the number of manual requesting gestures. This suggests that chimpanzees used higher manual gestures to request distant pieces of food. Importantly, note that only one piece of food was presented at a time so the chimpanzees did not have to encode distance information, and the virtual lines estimated from the angle of the hand (during both pointing and begging) did not seemingly aim at the food location. Taken together, these observations suggest that the height differentiation of the requesting gestures might be symbolic rather than explained by the geometric properties of the manual gestures. Furthermore, to make sure that the chimpanzees’ posture (sitting versus standing) did not influence the height of the hand (with, for instance, more standing for the distant piece of food, resulting in higher manual gestures), 20% of the trials (randomly chosen) were further coded. We observed that only one out of the eight chimpanzees was standing and that in only 0.64% of the “far” trials and 1.28% of the “near” trials. Figure *2c* (in the main text) shows the mean number of small and large mouth openings per trial depending on the distance of the food (“near” vs. “far”). The chimpanzees produced more large mouth openings when the food was far rather than near (GLMM, Z = 3.819, P < 0.001) but also more large mouth openings than small mouth openings when the food was far (Z = 5.222, P < 0.001). This effect was not significant for the small mouth openings or when the food was close, which suggests that, for a close referent, manual distance encoding is sufficient whereas chimpanzees might need to provide more information for farther referents, likely using the oral system. It is possible that this distance encoding mechanism would rely on particular combinations of manual and oral signals.

**References**

1. Watanuki K, Ochiai T, Hirata S, Morimura N, Tomonaga M, Idani G, Matsuzawa T. 2014 Review and long-term survey of the status of captive chimpanzees in Japan in 1926–2013. *Primate Res*. **30**, 147–156 (Japanese text with English abstract). (doi: 1.2345/psj.30.009)

2. Matsuzawa T. 2006 Socio–cognitive development in chimpanzees: A synthesis of laboratory work and fieldwork. In *Cognitive development in chimpanzees* (eds. T Matsuzawa, M Tomonaga, M Tanaka), pp. 3–33. Tokyo, Japan: Springer-Verlag.

3. Tomonaga M. 2001 Investigating visual perception and cognition in chimpanzees (*Pan troglodytes*) through visual search and related tasks: From basic to complex processes. In *Primate Origin of Human Cognition and Behavior* (ed. T Matsuzawa), pp. 55–86. Tokyo, Japan: Springer-Verlag.

4. Matsuzawa T, Tomonaga M, Tanaka M. (eds.). 2006 *Cognitive development in chimpanzees*. Tokyo, Japan: Springer.

5. Hattori Y, Tomonaga M, Fujita K. 2011 Chimpanzees (Pan troglodytes) show more understanding of human attentional states when they request food in the experimenters hand than on the table.  Interaction Studies. **12**, 418–429. (doi: [10.1075/is.12.3.03hat](http://dx.doi.org/10.1075/is.12.3.03hat))

6. Kemmerer D. 1999 "Near" and "far" in language and perception. *Cognition*, **73(1)**, 35–63. (doi: 10.1016/S0010-0277(99)00040-2)

7. Leavens DA, Hopkins WD. 1998 Intentional communication by chimpanzees: A cross-sectional study of the use of referential gestures. *Dev Psychol.* **34(5)**, 813–822. (doi: [10.1037/0012-1649.34.5.813](http://psycnet.apa.org/doi/10.1037/0012-1649.34.5.813))

8. Kendon A. 1980 Gesticulation and speech: Two aspects of the process of utterance. In *The relationship of verbal and nonverbal communication* (ed. MR Key), pp. 207–227. The Hague: Mouton.

9. Ihaka R, Gentleman R. 1996 R: A language for data analysis and graphics. *J. Comp. Graph. Stat*. **5(3)**, 299–314. (doi: 10.2307/1390807)

10. Bates D, Mächler M, Bolker B, Walker S. 2014 Fitting linear mixed-effects models using lme4. *J. Stat. Softw*. **67(1)**, 1–48. (doi: 10.18637/jss.v067.i01)

11. Gonseth C. 2013 *Multimodalité de la communication langagière humaine: Interaction geste/parole et encodage de la distance dans le pointage* (Doctoral dissertation). Retrieved from theses.fr database. (Accession number 2013GRENS011)
